# A novel nutritional index as a predictor of mortality in dilated cardiomyopathy: a retrospective study

**DOI:** 10.7717/peerj.12704

**Published:** 2022-01-19

**Authors:** Can Wang, Yali Qing, Wuxian Chen, Gaoye Li

**Affiliations:** Cardiology, The First Affiliated Hospital of Guangxi Medical University, Nanning, Guangxi, China

**Keywords:** Dilated cardiomyopathy, Nutritional index, Biomarker, Prognosis

## Abstract

**Background:**

Research has associated nutritional status with the prognosis of cardiovascular diseases. This study aimed to investigate the prognostic value of a novel nutritional index, triglycerides × total cholesterol × body weight index (TCBI), in patients with dilated cardiomyopathy (DCM).

**Methods:**

This retrospective cohort study enrolled 445 patients with DCM. The median follow-up period was 2.8 years, and the primary endpoint was all-cause death.

**Results:**

During follow-up, the all-cause mortality was observed in 135 out of 445 patients (30.3%). In Kaplan–Meier survival analysis, the third TCBI tertile had a lower mortality risk (T3 *vs.* T2 *vs.* T1: 16.9% *vs.* 35.1% *vs.* 38.9%; log-rank *P* < 0.001). In the multivariable Cox regression analysis, patients in the third tertile were associated with a decreased mortality, whereas there was no significant difference between the T2 and T1 groups. Moreover, TCBI could significantly improve risk stratification (continuous net reclassification improvement and integrated discrimination improvement) over the Geriatric Nutritional Risk Index (GNRI) and N-terminal pro-brain natriuretic peptide (NT-proBNP).

**Conclusions:**

TCBI is independently associated with long-term survival in patients with DCM. Combination of TCBI and other biomarkers, such as GNRI and NT-proBNP, can significantly improve prognostic prediction. Further studies with larger sample size are required to validate our results.

## Introduction

Heart failure (HF) is the leading cause of death worldwide (Savarese & Lund, 2017; Tomasoni et al., 2020). Dilated cardiomyopathy (DCM) is one of the most common cause of HF with an estimated prevalence of approximately 1: 2500 in the general population and an incidence of 7/100,000/year (Taylor, Carniel & Mestroni, 2006). DCM, characterized by left ventricular dilatation and contractile dysfunction in the absence of significant coronary artery disease (CAD), hypertension, valvular and congenital heart disease, has a spectrum of genetic and nongenetic causes (Jefferies & Towbin, 2010; Reichart et al., 2019). Given the poor clinical outcomes of DCM (survival was approximately 70%–75% at 1 year and 50% at 5 years) (Dec & Fuster, 1994), identification of novel prognostic factors is necessary and useful in risk stratification and guiding individual treatment.

Malnutrition and cachexia are commonly seen among HF patients, which may result from chronic inflammation, metabolic disturbances, low nutritional intake, and other mechanisms (Rahman et al., 2016; Sciatti et al., 2016). Some simplified nutritional screening tools such as the Geriatric Nutritional Risk Index (GNRI), the prognostic nutritional index (PNI), and the Controlling Nutritional Status (CONUT), have been found to predict the outcome of heart failure (Lin et al., 2016). Recently, a novel nutritional index, triglycerides × total cholesterol × body weight index (TCBI), has been proposed to be a useful prognostic indicator in patients with CAD (Doi et al., 2018; Kim et al., 2021), acute decompensated heart failure (Ishiwata et al., 2020), and critically ill patients (Minami-Takano et al., 2019). However, in patients with DCM, the prognostic value of TCBI has not yet been studied. Thus, this study aimed to investigate the predictive value of TCBI for all-cause mortality in patients with DCM.

### Methods

### Study population

**Figure 1:**
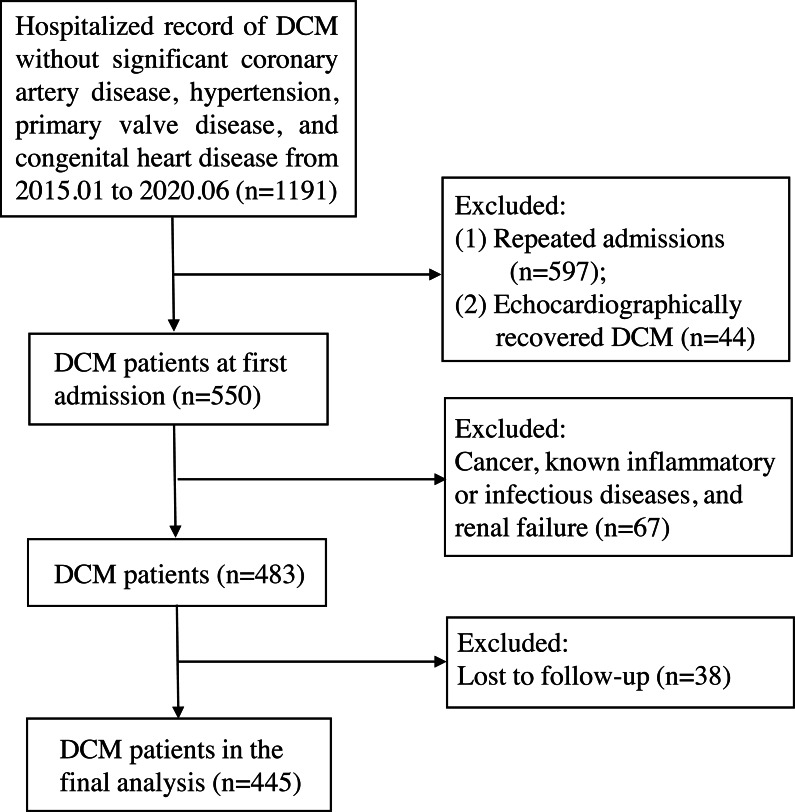
The study flow chart. The inclusion criteria were dilated cardiomyopathy without significant coronary artery disease, hypertension, primary valve disease, and congenital heart disease. After excluding patients with repeated admission, echocardiographically recovery after therapy (left ventricular end-diastolic diameter ≤ 5.0 cm for female or ≤ 5.5 cm for male, or left ventricular ejection fraction ≥ 45% and left ventricular fractional shortening ≥ 25%), cancer, known inflammatory or infectious diseases, renal failure, and loss to follow-up, 445 subjects were finally enrolled. DCM, dilated cardiomyopathy.

This retrospective cohort study enrolled 445 patients with discharge diagnosis of DCM at first admission to the First Affiliated Hospital of Guangxi Medical University between January 2015 and June 2020. DCM was defined by (a) the presence of left ventricle dilation with left ventricular end-diastolic diameter (LVEDD) > 5.0 cm (female) or > 5.5 cm (male); (b) left ventricular ejection fraction (LVEF) < 45% and left ventricular fractional shortening (LVFS) < 25%; and (c) the exclusion of significant CAD, hypertension, primary valve disease, and congenital heart disease. In addition, we excluded patients with cancer, known inflammatory or infectious diseases, and renal failure. The detailed study flow chart is shown in Fig. 1. The primary endpoint of the study was all-cause mortality. Follow-up outcomes were obtained from hospital medical records or telephone interviews, and follow-up time ended on June 2021. The study protocol was approved by the Human Research Ethics Committee of the First Affiliated Hospital of Guangxi Medical University, China (NO.:2021-KY-E-158) and fulfilled all principles of the Declaration of Helsinki. Given the retrospective study design, no informed consent was required.

### Relevant definition

Body weight, height and the laboratory data were all reviewed from the first admission record with DCM discharge diagnosis. Body mass index (BMI) was calculated as weight in kilograms divided by height in meters squared. Diabetes mellitus was defined as fasting glucose ≥7.0 mmol/l, glycated hemoglobin (A1C) ≥6.5%, or self-reported history of diabetes mellitus.

GNRI = [1. 489 ×albumin (g/L)] + 41.7× (measured body weight (kg)/ideal body weight (kg)] (Bouillanne et al., 2005) Ideal body weight = height (cm) −100 − [(height (cm) − 150)/4]c for men, or height (cm) –100 − [height (cm) − 150)/2.5] for women (Bouillanne et al., 2005).

PNI = serum albumin (g/L)+ (0.005 × total lymphocyte count in mm^3^).

TCBI = triglycerides (mg/dL) × total cholesterol (mg/dL) × body weight (kg)/1000.

CONUT was calculated based on serum albumin concentration, cholesterol level, and lymphocyte count with different point values assigned to various ranges of the laboratory measures (Ignacio de Ulíbarri et al., 2005).

### Statistical analysis

Continuous variables were described as mean ± standard deviation (SD) (for normal distributions) or median (interquartile range) (for skewed distributions), and categorical variables were presented as counts (percentages). The TCBI was stratified in tertiles: <701 (Q1), 701–1212 (Q2) and >1212 (Q3), and differences in baseline characteristics of the three groups were assessed by One-way ANOVA, Kruskal–Wallis test, chi-squared test, or Fisher’s exact test as appropriate. Spearman rank correlation was performed to evaluate the association between TCBI and other nutritional indexes. The prognostic value of TCBI was determined using Kaplan–Meier survival analysis with Log-rank test and Cox proportional hazards regression analysis. To assess the prognostic independence of TCBI, we created multivariable Cox regression models. Confounders were selected based on the clinical judgement and *P* ≤ 0.05 in the univariate analysis. Model 1 was adjusted for age and sex. Model 2 was adjusted for age, sex, and the variables in MAGGIC (Meta-Analysis Global Group in Chronic Heart Failure) risk score (Pocock et al., 2013), including BMI, current smoking, diabetes, symptom duration, systolic blood pressure, New York Heart Association (NYHA) class, LVEF, creatinine, use of beta–blocker, and use of angiotensin–converting enzyme inhibitor/angiotensin receptor blockers (ACEI/ARBs). Model 3 was adjusted for age, sex, and the other potential confounders in this study, including prior stroke, hemoglobin, neutrophils, albumin, uric acid, Na+, high-density lipoprotein cholesterol, low-density lipoprotein cholesterol, lipid–lowering drugs, LVEDD, and N-terminal pro-brain natriuretic peptide (NT-proBNP). To evaluate the predictive efficiency of different nutritional indexes, we compare the time-dependent area under the receiver operating characteristic (ROC) curve (AUC) using the R package ‘timeROC’. Also, multivariable Cox regression with the forward stepwise selection was built to investigate which nutritional indexes had independent prognostic value. To investigate the added prognostic value of TCBI beyond GNRI or NT-proBNP, C-index, continuous net reclassification improvement (NRI), and integrated discrimination improvement (IDI) at 3 years were calculated using the R packages‘survC1’ and‘survIDINRI’. All analysis was conducted with the use of R statistical software version 4.0.3 (R Foundation for Statistical Computing, Vienna, Austria). A two-tailed *P* value <0.05 was regarded statistically significant.

## Results

### Baseline characteristics

Baseline clinical characteristics according to the tertiles of TCBI are shown in Table 1. Of note, lower tertile groups of TCBI were likely to be older, female, and had lower BMI, blood pressure, lymphocyte, hemoglobin, albumin, Na+, left ventricular end-diastolic diameter, and a lower frequency of ACEI/ARBs treatment. In addition, lower tertile groups of TCBI had higher levels of NT-proBNP, NYHA class, and a higher frequency of hypolipemia.

**Table 1:** Patients characteristics.

	Total (*n* = 445)	TCBI T1 (*n* = 149)	TCBI T2 (*n* = 148)	TCBI T3 (*n* = 148)	*P*–value
Age, (years)	54.0 ± 13.5	56.6 ± 14.7	53.9 ± 13.2	51.5 ± 12.1	0.005
Male gender, n (%)	331 (74%)	99 (66%)	117 (79%)	115 (78%)	0.024
BMI, kg/m^2^	23.1 ± 3.8	21.5 ± 3.1	22.6 ± 3.4	25.2 ± 4.0	<0.001
Current smoking, n (%)	189 (42%)	55 (37%)	67 (45%)	67 (45%)	0.243
Diabetes, n (%)	129 (29%)	44 (30%)	34 (23%)	51 (35%)	0.092
Atrial fibrillation, n (%)	98 (22%)	42 (28%)	31 (21%)	25 (17%)	0.059
Prior stroke, n (%)	41 (9%)	19 (13%)	15 (10%)	7 (5%)	0.051
Symptom duration, n (%)					0.101
<1 year	172 (39%)	48 (32%)	54 (37%)	70 (47%)	
1–5 years	221 (50%)	83 (56%)	76 (51%)	62 (42%)	
>5 years	52 (12%)	18 (12%)	18 (12%)	16 (11%)	
Previous ICD/CRT, n (%)	22 (5%)	9 (6%)	9 (6%)	4 (3%)	0.306
Heart rate at admission, beats/min	89.3 ± 19.2	89.4 ± 21.0	89.4 ± 18.7	89.1 ± 18.0	0.985
Systolic blood pressure (mmHg)	113.6 ± 17.7	112.2 ± 17.8	109.9 ± 17.0	118.8 ± 17.2	<0.001
NYHA class, n (%)					0.028
I	16 (4%)	6 (4%)	4 (3%)	6 (4%)	
II	94 (21%)	19 (13%)	31 (21%)	44 (30%)	
III	167 (37%)	63 (42%)	53 (36%)	51 (35%)	
IV	168 (38%)	61 (41%)	60 (41%)	47 (32%)	
LVEDD (mm)	69.7 ± 8.4	68.2 ± 8.5	70.6 ± 8.1	70.2 ± 8.5	0.031
LVEF (%)	32 (27, 38)	30(26–38)	33 (26–37)	33 (29–39)	0.074
Laboratory tests at admission					
Hemoglobin (g/L)	134.9 ± 18.7	130.9 ± 18.8	135.3 ± 19.6	138.6 ± 16.7	0.001
Neutrophils (×10^9^/L)	5.1 ± 2.4	4.8 ± 2.3	5.0 ± 2.4	5.3 ± 2.5	0.137
Lymphocyte (×10^9^/L)	1.8 (1.4–2.3)	1.6 (1.2–1.9)	1.7(1.3–2.2)	1.9 (1.5–2.5)	<0.001
Albumin (g/L)	38.7 ± 4.7	37.3 ± 4.3	38.6 ± 4.7	40.2 ± 4.8	<0.001
Creatinine (umol/L)	92.0 (78.0–109.0)	91.0 (77.0–110.5)	93.0 (77.3–110.8)	90.0 (79.0–106.8)	0.938
Uric acid (umol/L)	506.2 ± 170.9	500.2 ± 165.2	503.5 ± 179.5	514.9 ± 168.7	0.739
Na^+^ (mmol/L)	138.3 ± 4.0	137.4 ± 4.6	138.6 ± 3.8	139.0 ± 3.3	0.001
K^+^ (mmol/L)	4.1 ± 0.6	4.1 ± 0.6	4.0 ± 0.6	4.1 ± 0.5	0.225
Total cholesterol (mg/dl)	168.9 ± 44.3	136.8 ± 28.6	167.7 ± 32.4	202.5 ± 43.2	<0.001
Triglyceride (mg/dl)	93.0 (72.6–121.3)	69.1 (58.5–75.3)	93.0 (81.5–107.2)	146.1 (115.6–191.8)	<0.001
HDL cholesterol (mg/dl)	38.8 ± 13.5	35.7 ± 12.4	38.41 ± 15.0	42.17 ± 12.1	<0.001
LDL cholesterol (mg/dl)	105.9 ± 34.3	83.31 ± 23.8	107.6 ± 25.9	126.80 ± 36.7	<0.001
NT–proBNP (pg/ml)	3914 (1856–8350)	6445 (3444–12578)	4045 (1948–8740)	2269 (1116–4896)	<0.001
Medications at discharge, n (%)					
Beta–blocker	396 (89%)	131 (88%)	131 (89%)	134 (91%)	0.751
ACEI/ARBs	362 (81%)	114 (77%)	117 (79%)	131 (89%)	0.020
Diuretics	438 (98%)	146 (98%)	146 (99%)	146 (99%)	1.000
Digoxin	344 (77%)	113 (76%)	117 (79%)	114 (77%)	0.800
lipid–lowering drugs	137 (31%)	25 (16.8)	41 (27.7)	71 (48.0)	<0.001
Nutritional index					
TCBI	907 (611–1402)	503 (420–611)	908 (811–1034)	1894 (1412–2604)	<0.001
GNRI	101 ± 10	96 ± 9	99 ± 9	107 ± 9	<0.001
PNI	48 ± 8	46 ± 10	48 ± 6	50 ± 7	<0.001
CONUT	2.0 (1.0–3.0)	3.0 (2.0–4.0)	1.5 (1.0–3.0)	1.0 (0.0–1.0)	<0.001

**Notes.**

Abbreviations ACEI angiotensin-converting enzyme inhibitor ARB angiotensin receptor blocker BMI body mass index CRT cardiac resynchronization therapy HDL high-density lipoprotein ICD implantable cardioverter defibrillator LDL low-density lipoprotein LVEF left ventricular ejection fraction NT-proBNP N-terminal pro brain natriuretic peptide NYHA New York Heart Association LVEDD left ventricular end-diastolic diameter

### Association between TCBI and other nutritional indexes

Spearman correlation analysis showed that TCBI were positively correlated with GNRI (r = 0.461, *P* < 0.001), PNI (r = 0.322, *P* < 0.001), but negatively correlated with CONUT (r = −0.516, *P* < 0.001).

### Kaplan–Meier survival and Cox regression analysis

**Figure 2:**
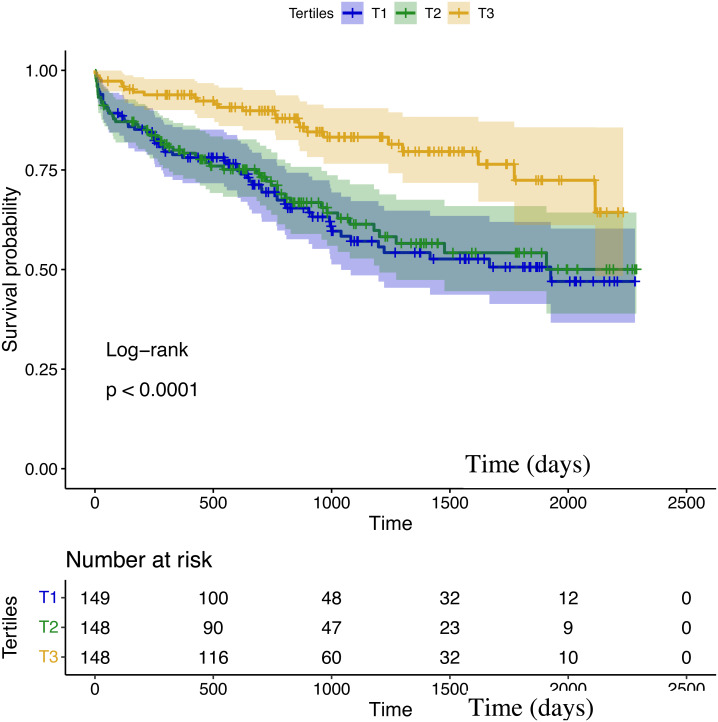
Kaplan–Meier survival curves in patients grouped according to TCBI tertiles.

During a median follow-up duration of 2.8 years, the all-cause mortality was observed in 135 out of 445 patients (30.3%). In Kaplan–Meier analysis, the third TCBI tertile had a lower mortality risk (T3 *vs.* T2 *vs.* T1: 16.9% *vs.* 35.1% *vs.* 38.9%; log-rank *P* < 0.001; Fig. 2).

All the variables with *P* ≤ 0.05 in univariable Cox regression analysis were shown in Table 2. Of note, each 1-SD increase in TCBI was associated with a lower risk of death (hazard ratio [HR]: 0.54, 95% confidence interval [CI] [0.39–0.75]; *P* < 0.001). The risk remained significant after adjustment of age and sex in model 1 (HR per 1-SD increase: 0.54, 95% CI [0.38–0.75]; *P* < 0.001), after adjustment of the predictors in MAGGIC risk score in model 2 (HR per 1-SD increase: 0.65, 95% CI [0.46–0.92]; *P* = 0.015), and after adjustment of the other potential confounders in model 3 (HR per 1-SD increase: 0.59, 95% CI [0.40–0.88]; *P* = 0.010) (Table 3). Moreover, when TCBI was categorized into tertiles, patients in the third tertile were associated with a decreased mortality, whereas there was no significant difference between the T2 and T1 groups (Table 3).

**Table 2:** Cox regression in univariable analysis for predicting mortality.

	Univariable	
	HR (95% CI)	*P* value
BMI, per 1 kg/m^2^	0.93 (0.89–0.98)	0.003
Prior Stroke	1.82 (1.12–2.96)	0.016
Symptom duration, per 1 year	1.05 (1.01–1.09)	0.022
Systolic blood pressure, per 10 mmHg	0.79 (0.71–0.88)	*P* < 0.001
NYHA class, per 1 class	1.39 (1.12–1.73)	0.003
LVEDD, per 10 mm	1.49 (1.25–1.79)	*P* < 0.001
LVEF, per 10%	0.79 (0.64–0.98)	0.032
Neutrophils, per 1 ×10^9^/L	1.07 (1.01–1.15)	0.032
Albumin, per 1 g/L	0.94 (0.91–0.98)	0.002
Creatinine, per 10 umol/L	1.01 (0.99–1.03)	0.083
Uric acid, per 10 umol/L	1.20 (1.09–1.32)	*P* < 0.001
Na^+^, per 10 mmol/L	0.53 (0.39–0.72)	*P* < 0.001
HDL cholesterol, per 10 mg/dl	0.81 (0.70–0.93)	0.003
LDL cholesterol, per 10 mg/dl	0.95 (0.90–0.99)	0.041
NT-proBNP, per 100 pg/ml	1.06 (1.04–1.07)	*P* < 0.001
ACEI/ARBs	0.51 (0.35–0.74)	*P* < 0.001
Nutritional index		
TCBI, per 1 SD	0.54 (0.39–0.75)	*P* < 0.001
GNRI, per 1 SD	0.69 (0.58–0.82)	*P* < 0.001
PNI, per 1 SD	0.78 (0.63–0.96)	0.020
CONUT, per 1 SD	1.31 (1.14–1.51)	*P* < 0.001

**Notes.**

Abbreviations ACEI angiotensin-converting enzyme inhibitor ARB angiotensin receptor blocker BMI body mass index CI confidence interval HDL high-density lipoprotein HR hazard ratio LDL low-density lipoprotein LVEDD left ventricular end-diastolic diameter LVEF left ventricular ejection fraction LVESD left ventricular end-systolic diameter NT-proBNP N-terminal pro brain natriuretic peptide NYHA New York Heart Association SD standard deviation

**Table 3:** Multivariable Cox regression analysis of TCBI for predicting mortality.

	Model 1		Model 2		Model 3	
	HR (95% CI)	*P* value	HR (95% CI)	*P* value	HR (95% CI)	*P* value
Each 1–SD increase in TCBI	0.54 (0.38–0.75)	<0.001	0.65 (0.46–0.92)	0.015	0.59 (0.40–0.88)	0.010
Tertiles of TCBI						
T1	Reference	/	reference	/	Reference	/
T2	0.92 (0.63–1.34)	0.648	0.93 (0.63–1.38)	0.729	0.95 (0.62–1.46)	0.817
T3	0.38 (0.24–0.61)	<0.001	0.53 (0.32–0.90)	0.018	0.47 (0.26–0.83)	0.010

**Notes.**

Model 1 was adjusted for age and sex; Model 2 was adjusted for variables in model 1 as well as BMI, current smoking, diabetes, symptom duration, systolic blood pressure, NYHA class, LVEF, creatinine, use of beta–blocker, and use of ACEI/ARBs. Model 3 was adjusted for variables in model 1 as well as prior stroke, hemoglobin, neutrophils, albumin, uric acid, Na+, HDL cholesterol, LDL cholesterol, lipid–lowering drugs, LVEDD, and NT–proBNP. CI, confidence interval; HR, hazard ratio; SD, standard deviation.

### Comparison between TCBI and other nutritional indexes for predicting mortality

**Figure 3:**
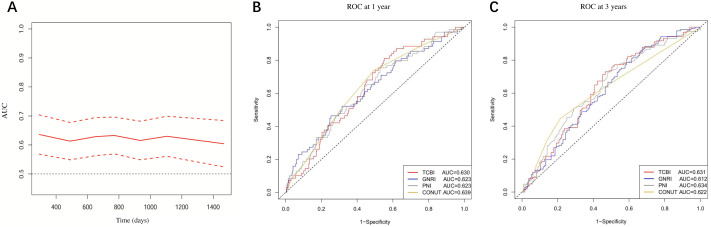
(A) Time-dependent AUC of TCBI; (B) and (C) AUC of TCBI, GNRI, PNI, and CONUT at 1 and 3 years, respectively.

The time-dependent AUC of TCBI for predicting mortality was shown in Fig. 3A. At 1 year, the AUC of TCBI, GNRI, PNI and CONUT were 0.630, 0.623, 0.623, and 0.639 (all *P* < 0.01) respectively (Fig. 3B). At 3 years, the AUC of TCBI, GNRI, PNI and CONUT were 0.631, 0.612, 0.634, and 0.622 (all *P* < 0.01) respectively (Fig. 3C). There were no statistically significant differences between TCBI and other nutritional indexes for prognostic prediction at 1 or 3 years (AUC comparison, all *P* > 0.05). In multivariable Cox regression including the four nutritional indexes, only TCBI (HR per 1-SD increase: 0.64, 95% CI [0.46–0.90]; *P* = 0.009) and GNRI (HR per 1-SD increase: 0.78, 95% CI [0.65–0.95]; *P* = 0.013) were independent predictor of mortality after the forward stepwise selection, suggesting that TCBI and GNRI might be the better predictors of mortality. We next investigated whether TCBI could significantly improve the prediction over GNRI. The addition of TCBI to GNRI showed no improvements in C-index (0.639 *vs.* 0.615; *P* = 0.134). However, TCBI significantly improved continuous NRI (NRI: 0.161, 95% CI [0.023–0.290]; *P* = 0.027) and IDI (IDI: 0.020, 95% CI [0.002–0.048]; *P* = 0.020) over GNRI.

### Incremental prognostic value of TCBI over NT-proBNP

Considering NT-proBNP as a classic prognostic biomarker of HF, we investigated whether TCBI could significantly improve the prediction of all-cause death over NT-proBNP. The addition of TCBI to NT-proBNP was not associated with a significant improvement in the C-index for prognostic prediction (0.691 *vs.* 0.679; *P* = 0.362). However, TCBI significantly improved continuous NRI (NRI: 0.189, 95% CI [0.050–0.305]; *P* = 0.006) and IDI (IDI: 0.024, 95% CI [0.005–0.053]; *P* = 0.008) over NT-proBNP.

## Discussion

The present retrospective study for the first time investigated the prognostic value of TCBI in DCM, and we found that TCBI is independent predictor of all-cause mortality. In addition, TCBI can significantly improve prognostic prediction over GNRI and NT-proBNP.

In the past, DCM is characterized by a high mortality rate and a poor prognosis (survival was approximately 70%–75% at 1 year and 50% at 5 years) (Dec & Fuster, 1994). With the development of the effective pharmacological treatments and device therapies (implanted cardioverter–defibrillator and cardiac resynchronization therapy), the prognosis of DCM has been dramatically improved (Merlo et al., 2018). A study by Merlo et al. (2014) even reported a 5-year survival rate of 89% in DCM patients enrolled during 1998–2007. In our study, the mortality rate was 30.3% during a median follow-up of 2.8 years, which is relatively high. The reasons for this were unclear and possible explanations may include the following. First, the result might be influenced by the retrospective design and a 7.9% loss to follow-up. Second, the device therapies in our cohort are considerably low. Third, our study exclude the DCM patients who were echocardiographically recovered after optimal therapies (LVEDD ≤ 5.0 cm for female or ≤ 5.5 cm for male, or LVEF ≥ 45%).

Although obesity has been considered as a risk factor for the development of HF, numerous clinical evidences suggested that obesity is associated with the better outcomes in those with established HF, which is called as the obesity paradox (Donataccio, Vanzo & Bosello, 2021; Horwich, Fonarow & Clark, 2018). Our study also observed such a phenomenon linking higher BMI and better prognosis (BMI per 1 kg/m2 increase, HR = 0.93 in the univariate analysis, *P* = 0.003). Our stepwise regression analysis, TCBI and GNRI were independent predictors of mortality, indicated that they may give more information of the nutritional status. TCBI is a new nutritional index integrating body weight and blood lipids. Lower total cholesterol and triglyceride levels have been found to be paradoxically predictive of poor clinical outcomes in HF patients, even after adjusting statin usage and traditional risk factors (Greene et al., 2013; Horwich et al., 2008). One explanation is that circulating lipid levels are indicators of nutritional status and inflammation (Araújo et al., 2008; Vaduganathan et al., 2014). Another explanation is that right-sided HF may lead to the passive hepatic congestion and impaired cholesterol synthetic ability.

A vicious circle exists between DCM and malnutrition. On one hand, DCM can lead to malnutrition through gastrointestinal malabsorption, metabolic disturbances, and chronic inflammation (Rahman et al., 2016; Sciatti et al., 2016). On the other hand, poor nutritional status has been identified as the root cause of DCM (Marinescu & McCullough, 2011). In recent years, TCBI has been shown to be associated with HF outcomes. In hospitalized critical patients with mechanical circulatory support devices, TCBI was an independent predictor of all-cause mortality (Minami-Takano et al., 2019). In patients with acute decompensated heart failure, TCBI can improve the prognostic prediction over traditional risk factors such as hemoglobin and serum sodium level (Ishiwata et al., 2020). Similarly, our study contributes to the existing literature on the relation of TCBI with DCM outcomes.

GNRI is a well validated prognostic predictor in HF patients (Hirose et al., 2020; Li et al., 2021). However, its prognostic effect on DCM is not well established. Kim et al. (Kim et al., 2021) found that in patients with acute myocardial infarction, GNRI had significantly higher AUC than TCBI (AUC 0.753 *vs.* 0.659, *P* < 0.05) for predicting the adverse cardiovascular events, whereas we found no significant differences between GNRI and TCBI for prognostic prediction in DCM. It is worthy to note that patients with CAD, especially acute myocardial infarction, usually have hypoalbuminemia and hyperlipidemia. Considering that TCBI is a lipid-based index, its prognostic value may be compromised in CAD patients, which leads to a different observation result. Given the fact that GNRI have the information about albumin and ideal body weight that were not included in TCBI, we assumed that combined indicators might be more accurate than single indicators in predicting the outcome. Indeed, TCBI can improve continuous NRI and IDI over GNRI. Furthermore, TCBI can further increase predictive accuracy on top of NT-proBNP, which is a classic prognostic biomarker in HF. All these evidences suggest that TCBI is a novel nutritional biomarker, and combined strategies are imperative for risk stratification. If validated in large cohort studies, our finding would be of great clinical significance.

This study was limited by the small sample size. Although baseline data were collected as comprehensively as possible, we could not entirely rule out the residual unknown confounders. In addition, the detailed reasons for death were not collected, and the dynamic reexamination of laboratory indexes and nutritional status monitoring were not conducted in our study. More data and verification are required. Effective nutritional risk assessment and nutritional intervention are expected to be a standard component in HF management in the future.

## Conclusions

TCBI is independently associated with long-term survival in patients with DCM. The combination of TCBI and other biomarkers, such as GNRI and NT-proBNP, can significantly improve prognostic prediction. Further studies with larger sample size are required to validate our results.

## Supplemental Information

10.7717/peerj.12704/supp-1 Supplemental Information 1 Raw data

## Additional Information and Declarations

### Data Availability

The following information was supplied regarding data availability: All raw data are available in the Supplementary File.
